# High-Fat Diet-Induced Obesity Model Does Not Promote Endothelial Dysfunction via Increasing Leptin/Akt/eNOS Signaling

**DOI:** 10.3389/fphys.2019.00268

**Published:** 2019-03-20

**Authors:** Vanessa da Silva Rocha, Erick Roberto Gonçalves Claudio, Vitor Loureiro da Silva, Jóctan Pimentel Cordeiro, Lucas Furtado Domingos, Márcia Regina Holanda da Cunha, Helder Mauad, Thiago Bruder do Nascimento, Ana Paula Lima-Leopoldo, André Soares Leopoldo

**Affiliations:** ^1^Physiology and Biochemistry Laboratory, Department of Sports, Center of Physical Education and Sports, Federal University of Espírito Santo, Vitória, Brazil; ^2^Department of Physiological Sciences, Health Sciences Center, Federal University of Espírito Santo, Vitória, Brazil; ^3^Vascular Biology Center, Medical College of Georgia, Augusta University, Augusta, GA, United States

**Keywords:** obesity, high-fat diet, leptin, Akt, vascular reactivity

## Abstract

Experimental studies show that the unsaturated high-fat diet-induced obesity promotes vascular alterations characterized by improving the endothelial L-arginine/Nitric Oxide (NO) pathway. Leptin seems to be involved in this process, promoting vasodilation via increasing NO bioavailability. The aim of this study was to test the hypothesis that unsaturated high-fat diet-induced obesity does not generate endothelial dysfunction via increasing the vascular leptin/Akt/eNOS signaling. Thirty-day-old male *Wistar* rats were randomized into two groups: control (C) and obese (Ob). Group C was fed a standard diet, while group Ob was fed an unsaturated high-fat diet for 27 weeks. Adiposity, hormonal and biochemical parameters, and systolic blood pressure were observed. Concentration response curves were performed for leptin or acetylcholine in the presence or absence of Akt and NOS inhibitor. Our results showed that an unsaturated high-fat diet promoted a greater feed efficiency (FE), elevation of body weight and body fat (BF), and an adiposity index, characterizing a model of obesity. However, comorbidities frequently associated with experimental obesity were not visualized, such as glucose intolerance, dyslipidemia and hypertension. The evaluation of the endothelium-dependent relaxation with acetylcholine showed no differences between the C and Ob rats. After NOS inhibition, the response was completely abolished in the Ob group, but not in the C group. Furthermore, Akt inhibition completely blunted vascular relaxation in the C group, but not in the Ob group, which was more sensitive to leptin-induced vascular relaxation. L-NAME incubation abolished the relaxation in both groups at the same level. Although Akt inhibitor pre-incubation reduced the leptin response, group C was more sensitive to its effect. In conclusion, the high-unsaturated fat diet-induced obesity improved the vascular reactivity to leptin and does not generate endothelial dysfunction, possibly by the increase in the vascular sensitivity to leptin and increasing NO bioavailability. Moreover, our results suggest that the increase in NO production occurs through the increase in NOS activation by leptin and is partially mediated by the Akt pathway.

## Introduction

Obesity, a disease characterized by excess body fat (BF), is recognized as a global epidemic since it affects virtually all age groups and social classes in both developed and developing countries (Suastika, 2006; Engin, 2017). The most common causes of the occurrence of obesity include increased caloric food intake and physical inactivity, which lead to a positive calorie balance and, consequently, increased BF (Stein and Colditz, 2004). Adipose tissue is considered not only a passive organ that stores energy, but a paracrine and endocrine organ that releases several active substances, including tumor necrosis factor-α (TNF-α), interleukin-6 (IL-6), adipsin, angiotensinogen, adiponectin, resistin and leptin, which may contribute to endothelial dysfunction (Frühbeck et al., 2001).

Endothelial dysfunction associated with obesity has been considered an independent risk of developing cardiovascular diseases (Poirier et al., 2006; Acree et al., 2007; Kumanyika et al., 2008; Stapleton et al., 2008; Graupera and Claret, 2018). For instance, obese animal models induced by a high-energy diet (33% ground chow, 33% Nestle condensed milk, 7% sucrose, and 27% water) present impaired endothelium-dependent and independent relaxation in mesenteric arteries (Naderali et al., 2001). Moreover, Fatani et al. (2007) demonstrated that animals fed a high-saturated fat diet (45% fat) do not present any difference for vasoconstrictor responses to potassium chloride (KCl) or noradrenaline; however, the vascular relaxation in response to carbachol and sodium nitroprusside (SNP) is attenuated in mesenteric arteries, indicating that the high-saturated fat diet promotes vascular dysfunction. In addition, other authors found no difference in acetylcholine-induced endothelium-dependent relaxation in the aorta of mice and obese rabbits induced by a high-fat diet with 58–60% of fat calories (Mundy et al., 2007; Bruder-Nascimento et al., 2017) or standard rabbit chow with 10% added fat (Jerez et al., 2012), which apparently is sex independent.

The mechanisms and factors involved in the vascular disease associated with obesity are uncertain, especially in the vascular endothelium. Several factors, such as insulin resistance and dyslipidemia, have been cited as possible factors that are responsible for cardiovascular dysfunction in models of obesity (Antonopoulos and Tousoulis, 2017). Furthermore, hyperleptinemia induces vasodilation directly, producing and releasing nitric oxide (NO), which diffuses through cellular membranes (Maenhaut and Voorde, 2011). Therefore, this double behavior of leptin and the modulation of vascular tone may help explain why obesity is not always accompanied by high blood pressure (Lembo et al., 2000).

Leptin is an adipocyte-derived hormone, which is elevated in obese humans and rodents (Maffei et al., 1995). In addition to controlling energy stores, it is also involved in several other physiological processes, including vascular tone modulation, acting through specific leptin receptors (Ob-Rb type) located in the vascular endothelium (Leung and Kwan, 2008). Leptin binding to its receptor will activate phosphatidylinositol 3-kinase (PI3K), activating protein kinase B (Akt) and protein kinase A (PKA), which phosphorylates endothelial nitric oxide synthase (eNOS), resulting in the production of NO (Lembo et al., 2000; Vecchione et al., 2002).

Our laboratory has revealed that obese rats induced by an high-unsaturated fat diet (20% fat) show an increase of vascular L-arginine/NO pathway in order to protect the integrity of vascular function in obesity, possibly mediated by elevated levels of plasma leptin (Bruder-Nascimento et al., 2011). Thus, the aim of this study was to test the hypothesis that unsaturated high-fat diet-induced obesity does not generate endothelial dysfunction via increasing the vascular leptin/Akt/eNOS signaling.

## Materials and Methods

### Animals

Thirty-day-old male *Wistar* rats (≈150 g) were obtained from the animal facility of the Federal University of Espírito Santo. Rats were individually housed, at constant room temperature (24 ± 2°C), humidity (55 ± 5%) and light cycles (12-h light/dark), and had free access to water.

All experiments and procedures were performed in accordance with the Guide for the Care and Use of Laboratory Animals, published by the United States National Institutes of Health, and were approved by the Ethics Committee on Animal Use of the Federal University of Espírito Santo (protocol 027/2014).

### Experimental Design

Rats were randomly distributed into two groups: control (C, *n* = 20), which were fed standard rat chow (Presence chow, SP, Brazil); and obese (Ob, *n* = 18), which were fed a high-unsaturated fat diet (RC Focus 2413, Agroceres^®^, Rio Claro, SP, Brazil) for 27 weeks. The dietary ingredients used to prepare the high-fat diet were sodium chloride, casein, powdered milk, soybean protein concentrate, whole corn, cracker flour, dicalcium phosphate, calcium carbonate, additives emulsifier, antioxidants, and cheese flavoring, along with a vitamins and minerals mixture (Leopoldo et al., 2011). The animals received 50 g of feed daily, and after 24 h, food that was not ingested was weighed for food intake evaluation. The components and nutritional values are displayed in Table 1. The high-fat diet was high in calories (high-fat diet = 3.65 kcal/g vs. standard diet = 2.95 kcal/g) due to a higher fat composition made from saturated (20%) and non-saturated (80%) fatty acids. The nutritional status was determined by food consumption (FC), caloric intake (CI) and feed efficiency (FE). Calorie intake (CI) was calculated weekly by the average weekly FC (g) × dietary energetic density (kcal). FE was defined as the ability of animals to convert feed energy consumed in body weight and was measured by dividing body weight gain (g) by the total caloric intake (kcal) and multiplying by 100. In addition, body weight was recorded weekly.

**Table 1:** Components and nutritional values of diets.

	Experimental diets	
Components (%)	Control	High-fat
Protein	23.0	20.0
Carbohydrate	42.8	26.4
Fat	4.0	20.0
Mineral and vitamins	12.1	12.1
Fiber	5.1	9.0
Humidity	13.0	12.5
**Nutrient composition (%)**		
Energy from protein	30.7	21.9
Energy from carbohydrate	57.3	28.9
Energy from fat	12.0	49.2
**Energy density (Kcal/g)**	2.99	3.65


### Determination of Obesity

At the end of the experimental protocol, the fat index was used as an indicator of obesity. After 27 weeks of developing obesity, animals were anesthetized intraperitoneally by ketamine and xylazine (50 and 10 mg/kg, respectively) and decapitated. After median thoracotomy, fat pads of adipose tissue were dissected and weighed. This method allowed the accurate and consistent assessment of the amount ofBF. The adiposity index, used to assess obesity, was calculated with the following formula: adiposity index [BF/final body wt] × 100. BF was measured from the sum of the individual fat pad weights as follows: BF = epididymal fat + retroperitoneal fat + visceral fat.

### Constitution of the Control and Obese Groups

After 27 weeks of the experimental protocol, a 95% confidence interval (CI) was built for the adiposity index from the Ob and C rats and was adopted as the separation point (SP) between the groups, as previously described (Ferron et al., 2015). After the application of the criterion for determining obesity, seven animals from the control group and nine animals from the obese group were excluded. Thus, our study was carried out with 20 animals in the control group and 18 animals in the obese group.

### Comorbidities and Hormones Associated With Obesity

Obesity developed by a high-fat diet may have changes in the cardiovascular, metabolic and hormonal profile, such as hypertension, glucose intolerance, systemic insulin resistance, dyslipidemia, hyperglycemia, hyperinsulinemia, and hyperleptinemia. Thus, these were evaluated in all groups.

### Measurement of Systolic Blood Pressure (SBP)

Systolic blood pressure was determined by using the non-invasive tail-cuff plethysmography method (IITC INC/Life Science, Woodland Hills, CA, United States) in conscious rats. The rats were pre-warmed at 35 ± 2°C for 5 min and placed in a cylindrical acrylic tube. At the proximal region of the animal’s tail, a cuff was connected that was inflated to 200–250 mmHg; signals were sent and recorded in the computer to obtain pressure data. At least three measurements were performed for each animal to obtain an average record.

### Glucose Tolerance Test (GTT)

Following 27 weeks of treatment, all rats fasted for 6 h, and a blood sample from the tip of the tail of conscious rats was collected. The basal blood glucose level of each animal was immediately determined using a handheld glucometer (Accu-Check Go Kit; Roche Diagnostic Ltd., São Paulo, Brazil). Subsequently, an injection of glucose solution (2 g/kg body weight) dissolved in water was administered intraperitoneally, and blood glucose levels were measured after 15, 30, 60, 90, and 120 min. Homeostatic Model Assessment for Insulin Resistance (HOMA-IR) was calculated according to the following formula: fasting glucose (mmol/l) × fasting insulin (μU/ml)]/22.5.

### Biochemical and Hormonal Determination

For biochemical and hormonal analysis, animals fasted for 12–15 h, then were anesthetized with chloral hydrate (1.0 ml/100 g body wt, intraperitoneally) and sacrificed by decapitation. Blood was collected in Falcon tubes, centrifuged at 3,000 × g for 15 min and stored at -80°C. Serum levels of glucose, triacylglycerol (TG), total cholesterol (T-Chol), protein and high-density lipoprotein (HDL) were determined using a specific kit (Bioclin Bioquímica^®^, Belo Horizonte, Minas Gerais, Brazil) and analyzed by biochemical analyzer BS-200 (Mindray do Brasil – Comércio e Distribuição de Equipamentos Médicos Ltda, São Paulo, Brazil). Serum insulin and leptin levels were measured using an enzyme-linked immunosorbent assay (ELISA) using specific kits (Linco Research Inc., St. Louis, MO, United States). The reading was carried out using a microplate reader (Asys Expert Plus Microplate Reader, Cambourne, Cambridge, United Kingdom).

### Post-death Morphological Analysis

Animals were anesthetized with chloral hydrate, euthanized by decapitation and thoracotomized. At the end of the experimental protocol, the heart, ventricles and atrium, and tibia were separated, dissected, weighed and measured on a precision scale (AD 500, Mars Scientific and Industrial Instrumentation Ltd., Minas Gerais, Brazil). Cardiac remodeling at the macroscopic level, which identifies the presence or absence of cardiac hypertrophy, was determined by analyzing the following parameters: heart weight (HW), left and right ventricles (LV and RV) and atrium (AT) weights, HW, LV and RV/tibia length ratios.

### Aortic Vascular Reactivity

The animals were anesthetized with a mixture containing ketamine and xylazine (50 mg/kg and 10 mg/kg, respectively) and sacrificed by decapitation. The chest was opened, and the descending thoracic aorta artery was removed and immersed in a modified Krebs-Henseleit solution composed of (in mM/L) NaCl 118, KCl 4.7, MgSO_4_ 1.2, CaCl_2_ 1.6, K_2_HPO_4_ 1.2, NaHCO_3_ 25 and glucose 11, and aerated with a carbogenic mixture containing 5% CO_2_ and 95% O_2_. The connective and adipose tissues were removed, and the artery was divided into sections of approximately 3.5 mm in length. Each vascular ring was mounted in an isolated organ bath system (Panlab Organ Bath System, ADInstruments, Australia) containing 5 mL of Krebs-Henseleit solution heated to 37 ± 0.5°C and was continuously gassed with the carbogenic mixture. Changes in vessel diameter were recorded by a force transducer connected to a data acquisition system (Power Lab, ADInstruments, Australia). During the 45 min stabilization period, the aortic rings were subjected to a resting tension of approximately 1 g and adjusted when necessary. To test the functional integrity the rings were contracted with KCl 75 mM, and after washing and returning to the baseline values, the rings were contracted with 10^-7^ M phenylephrine and relaxed with 10^-5^ M acetylcholine to test the endothelial integrity.

The concentration-response constriction curve was obtained using increasing concentrations of phenylephrine (10^-10^ to 10^-4^ M), and the data was shown as a percentage of contraction corrected by the maximal contraction induced by KCl. Prior to the concentration-response relaxation curves, all the rings were pre-constricted with 10^-7^ M of phenylephrine. The endothelium-dependent relaxation curves with acetylcholine (10^-11^ to 10^-4^ M) and leptin (10^-14^ to 10^-6^ M) were obtained. To analyze the role of Akt and NO in response to leptin and acetylcholine, aortic rings were exposed to the 1L6-hydroxymethyl-chiro-inositol-2-(*R*)-2-*O*methyl-3-*O*-octadecyl-*sn*-glycerocarbonate “Akt inhibitor” (5 μM, Calbiochem, Merck, Darmstadt, Germany) and Nω-Nitro-L-arginine methyl ester (L-NAME, 100 μM, Sigma-Aldrich, United States) 30 min prior to the concentration-response curves. To evaluate the baseline NO production in the vascular constriction, the vessel rings of control and obese rats were exposed to L-NAME (100 μM) before the constrictor response curve to phenylephrine.

### Statistical Analysis

Data on general characteristics, nutritional assessment and comorbidities were reported as mean ± standard error of the mean (SEM) and submitted to the Kolmogorov-Smirnov test to determine adherence to normality. Comparisons of groups were analyzed by Student’s *t*-test for independent samples. A repeated measure of analysis of variance (ANOVA), followed by Tukey *post hoc* test, was performed to evaluate the change in body weight. In addition, the concentration-response curves were analyzed using two-way ANOVA, followed by Fisher *post hoc* tests. The *p*-value was considered significant when <5%.

## Results

### General Characteristics and Cardiac Morphology

**FIGURE 1:**
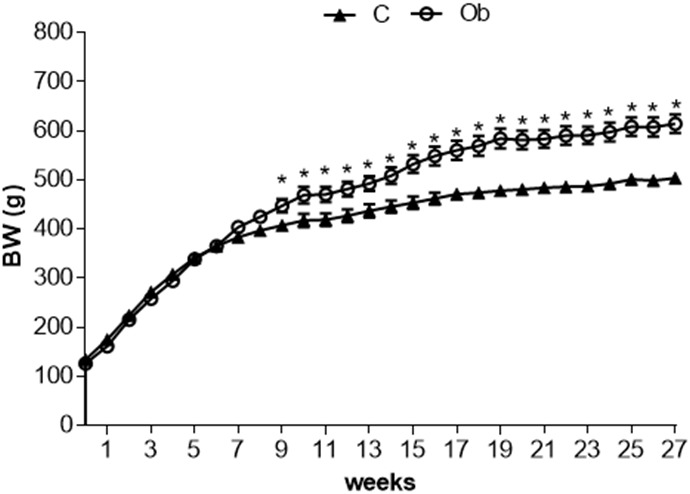
Evolution of body weight at 27 week of experimental protocol of obesity. C, control; Ob, obese; BW, body weight. Data are reported as the means ± SEM. Two-way ANOVA repeated measures for independent samples followed by Tukey *post hoc* test. ^∗^*p* < 0.05 Ob vs. C.

Figure 1 illustrates the significant separation of body weight between C and Ob groups observed at week 9, which remained significantly greater during the 27 weeks of the experimental protocol. Obesity induced by a high-fat diet had a substantial elevation in adiposity parameters, FE, LV, and LVW/tibia length when compared to C rats (Table 2). In contrast, the FC was lower in Ob than in C rats. In addition, there was no difference between the groups in relation to IBW, calorie intake and absolute heart, as well as heart/tibia length and tibia length (data not shown) (Table 2).

**Table 2:** General characteristics and cardiac morphology.

Variables	Groups	
	C (*n* = 20)	Ob (*n* = 18)
IBW (g)	134 ± 3	126 ± 3
FBW (g)	504 ± 11	614 ± 19*
Weight gain (g)	370 ± 11	488 ± 21*
Visceral fat (g)	8.2 ± 0.7	15.3 ± 1.5*
Retroperitoneal fat (g)	13.2 ± 0.9	32.2 ± 3.0*
Epididymal fat (g)	6.8 ± 0.3	11.2 ± 0.6*
Body fat (g)	28.2 ± 1.8	58.6 ± 4.7*
Adiposity index (%)	5.5 ± 0.3	9.3 ± 0.5*
FC (g/day)	22.8 ± 0.6	18.6 ± 0.3*
CI (g × kcal/day)	67.3 ± 1.7	67.9 ± 1.1
FE (%)	2.91 ± 0.05	3.82 ± 0.18*
Heart (g)	1.18 ± 0.05	1.25 ± 0.04
LVW (g)	0.83 ± 0.02	0.90 ± 0.03*
Heart/Tibia-length (g/cm)	0.28 ± 0.01	0.30 ± 0.01
LVW/Tibia-length (g/cm)	0.20 ± 0.01	0.22 ± 0.01*


*Values are means ± SEM. C, control; Ob, obese; IBW, initial body weight; FBW, final body weight; FC, food consumption; CI, calorie intake; FE, feed efficiency; LVW, left ventricle weight. Student’s *t*-test for independent samples. ^∗^*p* < 0.05 Ob vs. C.*

### Comorbidities Associated With Obesity

**FIGURE 2:**
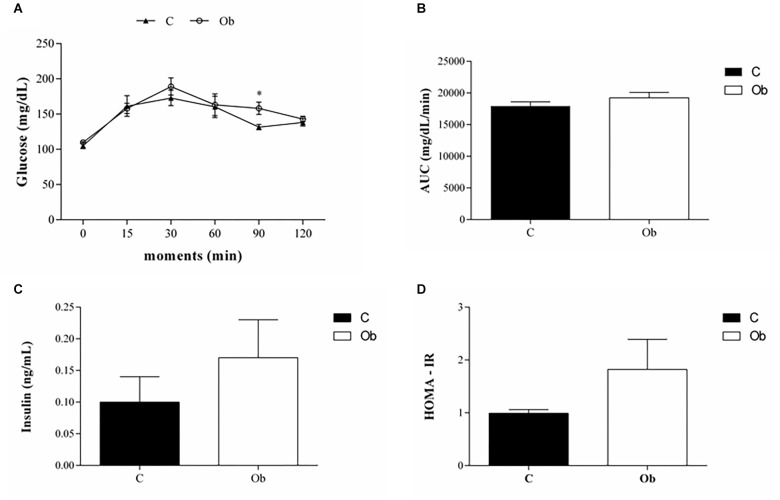
Glucose tolerance profile at 27 week of experimental protocol of obesity. Glucose tolerance test **(A)**, area under the curve for glucose – AUC **(B)**, insulin serum level **(C)** and **(D)** homeostatic model assessment index (HOMA-IR) from control (C; *n* = 9) and obese (Ob; *n* = 7) groups. Data are reported as the means ± SEM. Two-way ANOVA repeated measures for independent samples followed by Tukey *post hoc* test. ^∗^*p* < 0.05 Ob vs. C.

There were no significant differences in comorbidities associated with obesity, since the SBP, TG, Cholesterol, LDL, HDL, and protein were similar between the groups (Table 3). The glucose tolerance profile, AUC, insulin levels and HOMA-IR were also not affected by exposure to obesity (Figure 2). However, the Ob rats presented only higher levels of glucose at 90 min than the C rats (Figure 2A). In addition, the Ob group exhibited higher levels of leptin when compared to C (Table 3).

**Table 3:** Comorbidities associated with obesity.

Variables	Groups	
	C (*n* = 9)	Ob (*n* = 7)
SBP (mmHg)	122 ± 5	128 ± 6
TG (mg/dL)	76 ± 6	76 ± 10
Cholesterol (mg/dL)	78 ± 6	68 ± 4
LDL (mg/dL)	11.1 ± 1.4	11.3 ± 0.9
HDL (mg/dL)	20 ± 2	21 ± 2
Protein (g/dL)	5.36 ± 0.34	5.40 ± 0.28
Leptin (ng/mL)	0.77 ± 0.08	1.32 ± 0.21*


*Values are reported as the means ± SEM. C, control; Ob, obese; SBP, systolic blood pressure; TG, triglycerides; LDL, low-density lipoprotein; HDL, high-density lipoprotein. Student’s *t*-test for independent samples. ^∗^*p* < 0.05 vs. C.*

### Vascular Reactivity

**FIGURE 3:**
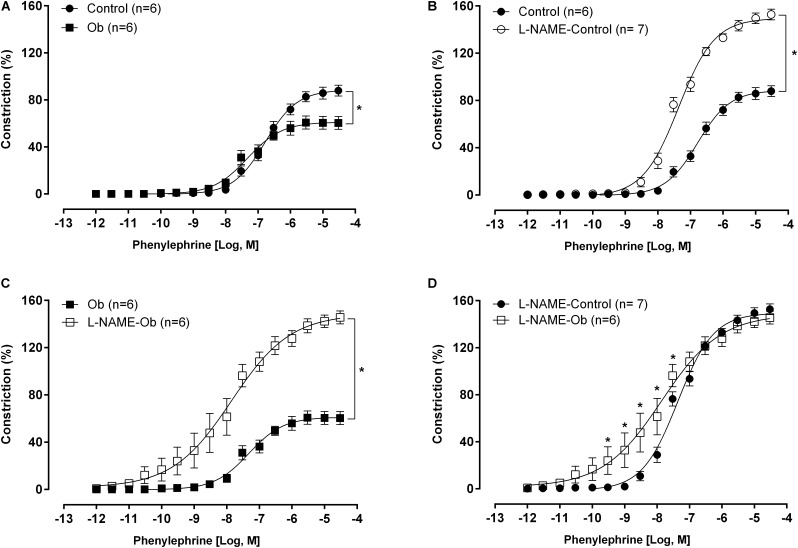
Concentration-response curve to phenylephrine obtained from control and obese (Ob) groups rings before **(A)** and after nitric oxide synthase inhibition with L-NAME (100 μM – **B–D**). Parentheses indicates the number of rings and rats, respectively. Data are presented as the mean ± SEM. Two-way ANOVA followed by the Fisher′s *post hoc* test for multiple comparisons. ^∗^*p* < 0.05.

The maximal contraction induced by 75 mM KCl stimulation was not different between groups (C: 3.25 ± 0.08 g vs. Ob: 3.05 ± 0.08 g; *p* > 0.05). The concentration-response curve to phenylephrine showed a significant reduction in the constrictor response obtained from vessels of Ob animals compared to C (Figure 3A). L-NAME increased the adrenergic response in both groups compared with its respective control curve (Figure 3B,C). However, Ob rats were more sensitive to L-NAME effects, and the phenylephrine curve from Ob rats presented a shift to the left compared to the C rats, but no difference in the maximum response (Figure 3D).

**FIGURE 4:**
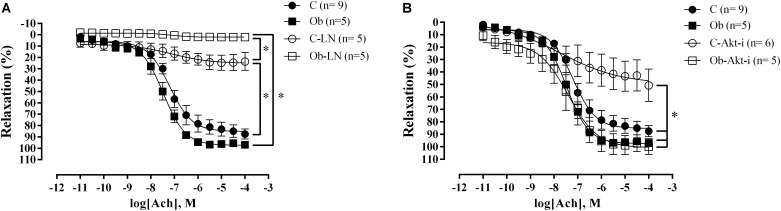
Concentration-response curve to acetylcholine in the thoracic aorta obtained from C and Ob groups in the presence or absence of L-NAME (100 μM – **A**) or Akt inhibitor (5 μM – **B**). Parentheses indicates the number of rings and rats, respectively. Data are presented as mean ± SEM. Two-way ANOVA followed by the Fisher′s *post hoc* test for multiple comparisons. ^∗^*p* < 0.05.

The endothelium-dependent relaxation with acetylcholine showed no differences between the C and Ob rats (Figure 4A). However, NO synthesis inhibition generated a significant reduction in relaxation for both groups, compared with their respective controls (Figure 4A). In addition, L-NAME completely abolished the vascular relaxation in the Ob group, and partially in the C group (Figure 4B). On the other hand, Akt inhibition promoted reduction in the acetylcholine response only in C rats (Figure 4B).

**FIGURE 5:**
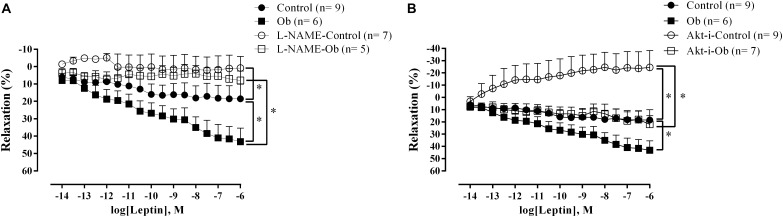
Concentration-response curve to leptin in the thoracic aorta from C and Ob groups in the presence or absence of L-NAME (100 μM – **A**) or Akt inhibitor (5 μM – **B**). Parentheses indicates the number of rings and rats, respectively. Data are presented as mean ± SEM. Two-way ANOVA was used followed by the Fisher′s *post hoc* test for multiple comparisons. ^∗^*p* < 0.05 Ob vs. C.

Vasodilation-induced by leptin showed a greater response in aortic rings from Ob rats compared to that of the C rats (Figure 5A). The presence of L-NAME blunted the response of leptin-induced relaxation in both groups (Figure 5A). On the other hand, Akt inhibition resulted in reduced vascular leptin response in both groups, but this effect was less pronounced in Ob rats compared to C rats, suggesting that leptin promoted vascular relaxation partially mediated by Akt activation in obesity (Figure 5B).

## Discussion

The main findings of the present study were as follows: (i) the high-fat diet led to the development of obesity; (ii) a reduction in the vasoconstrictor response to phenylephrine in obese animals; (iii) endothelial dysfunction was not observed in this model of obesity, since thoracic aorta from obese rats were still responsive to leptin effects; (iv) a significantly higher vasodilator response to leptin was observed in the obese group, which was abolished when exposed to NOS blockade in both groups; (v) there was a reduction in the vasodilator response to leptin in both groups, compared with the respective control, when the rings were exposed to Akt inhibitor.

Experimental models that mimic the eating habits of the human population have been widely used to elucidate the mechanisms of obesity and metabolic disorders (Angelova and Boyadjiev, 2013). In this sense, our data show that rats fed the high-fat diet developed obesity characterized by an increase in the FBW, total BF and fat pads (retroperitoneal, visceral and epididymal); however, these animals had hyperleptinemia frequently related and observed in the experimental and human studies with obesity (Woods et al., 2003; Nascimento et al., 2008). In addition, obesity is often associated with metabolic and endocrine disorders, such as glucose intolerance, insulin resistance, hyperleptinemia, dyslipidemia, and hypertension. Nevertheless, in this obesity model, no changes were observed in basal glucose, insulin or alterations of the lipid profile.

In addition to absence of changes in the metabolic abnormalities, the model proposed did not lead to an elevation in blood pressure levels. This finding corroborates studies that have verified a significant reduction in SBP in obese animals (Reilly and Rader, 2003; Carroll et al., 2006; Lima-Leopoldo et al., 2011). Some studies have shown that a high-unsaturated fat diet is related to the production of eNOS stimulated by hyperleptinemia and that it may be responsible for increased production and release of NO, resulting in a reduction of peripheral resistance and, consequently, lower blood pressure (Bruder-Nascimento et al., 2011, 2017).

The results from the post-death morphological analysis revealed obesity-induced cardiac hypertrophy, evidenced by elevation of absolute LV weight, as well as LV/tibia length. These data corroborate several studies that indicate obesity induced by a high-fat diet for 15 and 30 weeks promotes cardiac hypertrophy; these authors suggest that cardiac remodeling occurs due to the long period of obesity (Lima-Leopoldo et al., 2014; Silva et al., 2014), which is considered an adaptive characteristic of obesity (Rider et al., 2009). Thus, myocyte hypertrophy is considered a mechanism of adaptation in response to several stimuli, such as increased cardiac output due to the metabolic demand caused by obesity condition (Mill and Vassallo, 2001; Rahmouni et al., 2005).

Obesity constitutes a major cause of CVD, including hypertension, atherosclerosis, ischemic heart disease and heart failure. It is widely known that a high intake of saturated fat increases the risk of cardiovascular diseases, while diets enriched with unsaturated fatty acids seem to lower this risk (Lorente-Cebrián et al., 2013). Thus, several authors have shown beneficial effects on obesity-related vascular alterations (Bruder-Nascimento et al., 2011; Lorente-Cebrián et al., 2013; de Oliveira et al., 2017) In this sense, these alterations play a key role in the pathogenesis of these diseases, which includes the reduction of NO bioavailability as well as the elevation of components that induce vascular contractility, such as angiotensin-II and endothelin-1 (Zemel et al., 1990; Hopfner et al., 1998; Mundy et al., 2007). Furthermore, adiposity is associated with elevations in the adipocyte-derived hormone leptin, which causes a dual effect on the cardiovascular system. Centrally, it stimulates the sympathetic nervous system and increasing the arterial blood pressure, while peripherally, it modulates the release of several vasoactive factors, mainly NO, in the vascular endothelium (Kimura et al., 2000; Lembo et al., 2000).

Another effect of obesity induced by a diet rich in unsaturated fat for 27 weeks was the significant reduction in the vascular adrenergic response in Ob animals. This result was in accordance with our previously published results (Bruder-Nascimento et al., 2011, 2017). However, when the animals were submitted to the diet for 30 weeks, this difference was abolished (Bruder-Nascimento et al., 2011). In another study with obese mice induced by a high sugar diet for 8 weeks, Silva et al. (2016) verified a significant decrease in the contractile response to phenylephrine in obese animals that was associated with an increase in the vascular production of NO, which the authors attributed to the higher expression of iNOS. Moreover, in rabbits fed a hyperlipidemic diet for 12 weeks, hyporeactivity was observed in the responses stimulated by the noradrenaline and angiotensin-II (Jerez et al., 2012). Taken together, these results suggest that the time-exposure to the diet, and consequently to the obesity and hyperleptinemia, might be a determinant factor on vascular adaptations and the vasoconstrictor responses, which is mainly related to the adrenergic system.

On the other hand, Keita et al. (2013) and Bautista et al. (2014) evaluated the aortic vascular reactivity in rats with unsaturated hyperlipidemic diet-induced obesity (with fried canola oil and margarine) for 10 and 20 weeks, respectively, and demonstrated that obesity does not change the vasoconstrictor response to phenylephrine or noradrenaline. The responses to these vasoconstrictor agonists may be related to the amount and the nature of the lipids in the diet, which are considered protective factors and/or atherogenic and are associated with the modulation of NOS activity (Santos et al., 2013).

In our study, the lowest vasoconstrictor response observed in Ob animals occurred due to a higher basal production of NO, counterbalancing and reducing the constriction caused by the stimulation of adrenergic receptors. Furthermore, the vessels of Ob animals showed a higher dependency of NO in the endothelium-mediated relaxation since the response to acetylcholine and leptin were totally abolished when NOS was inhibited with L-NAME (Figure 4A, 5A). The higher basal production of NO is possibly associated with the increase in the plasmatic concentration of leptin in obese animals. Several mechanisms have been proposed for the increase in the NO production stimulated by leptin, which might be associated with a compensatory mechanism against its sympathetic stimulation (Mitchell et al., 2001). Lembo et al. (2000) and Kimura et al. (2000) verified that the relaxation induced by leptin occurs via production of endothelial NO in the rat aorta. In this sense (Vecchione et al., 2002). showed that leptin activates the Akt pathway by a mechanism independent of PI3K phosphorylation, increasing NO production by phosphorylating eNOS at the Ser1177 residue in the aorta of *Wistar Kyoto* rats. In addition, it was verified that insulin potentiates this response by increasing the Akt phosphorylation at the sites of Ser^473^ and Thr^308^ (Vecchione et al., 2003). In another study that evaluated the effect of leptin in human aortic endothelial cells, a new pathway was activated by leptin that was mediated by the α1 subunit of AMP-activated protein kinase (AMPK), which phosphorylates Akt at the Ser^473^ residue, promoting the activation of eNOS (Procópio et al., 2009). Recently, a study verified that in eNOS knockout mice, leptin and a fat-rich diet induced an increase in the endothelial nNOS expression by phosphorylating JAK-2/STAT-3, thereby compensating for the reduction of NO and restoring the endothelial function in angiotensin-II-infused mice (Benkhoff et al., 2012). We can verify that among many pathways described for leptin-induced NOS activation, most of them are triggered by Akt phosphorylation. However, according to our results, this pathway is partially activated in this model of obesity, since the Akt inhibition did not change the endothelium-mediated dilation with either acetylcholine or leptin to the same level as observed in C animals.

## Conclusion

In conclusion, the high-unsaturated fat (49.2 energy from fat) diet-induced obesity improved the vascular reactivity to leptin and does not generate endothelial dysfunction, possibly by the increase in the vascular sensitivity to leptin and increasing NO bioavailability. Moreover, our results suggest that the increase in NO production occurs through the increase in NOS activation by leptin and is partially mediated by the Akt pathway.

## Author Contributions

AL-L, VR, and AL participated and conceived the research design. EC, VdS, JC, LD, MdC, AL-L, and AL performed the experiments and data analysis. VR, EC, MdC, HM, TBdN, AL-L, and AL interpreted and discussed the data. AL, VR, EC, MdC, and AL wrote the manuscript. HM, TBdN, AL-L, and AL refined the final draft and revised the manuscript. All authors read and approved the final version of the manuscript.

## Conflict of Interest Statement

The authors declare that the research was conducted in the absence of any commercial or financial relationships that could be construed as a potential conflict of interest.
